# Review of Laboratory Tests used in Monitoring Hepatitis B Response to Pegylated Interferon and Nucleos(t)ide Analog Therapy

**DOI:** 10.1007/s40506-016-0080-x

**Published:** 2016-07-02

**Authors:** Carla Osiowy, Carla Coffin, Anton Andonov

**Affiliations:** 1 Bloodborne Pathogens and Hepatitis, National Microbiology Laboratory, Public Health Agency of Canada, 1015 Arlington St., Winnipeg, MB R3E 3R2 Canada; 2 Liver Unit, Division of Gastroenterology and Hepatology, Cumming School of Medicine, University of Calgary, Calgary, AB Canada

**Keywords:** Hepatitis B virus, Nucleos(t)ide analog, Peg-IFN, Antiviral therapy, qHBsAg, Monitoring response

## Abstract

There are only two currently approved classes of hepatitis B virus (HBV) antiviral agents, pegylated interferon (Peg-IFN), and nucleos(t)ide analogs (NAs) for chronic HBV infection. Although Peg-IFN is used for a finite 48-week duration and offers a greater chance of sustained off-treatment virological response, it is poorly tolerated and can only be offered to selected patients. The NAs are well tolerated but require prolonged therapy due to risk of relapse with treatment cessation. There is evolving data that novel virological assays (e.g., quantitative hepatitis B surface antigen, quantitative hepatitis B core antigen, quantitative antibody to core protein) in combination with hepatitis B genotype and more sensitive HBV DNA polymerase chain reaction (PCR) assays may be useful to predict response to IFN as well as off-treatment NA durability. Utilization of these clinical laboratory tests may be important given the development of novel anti-HBV therapies, hoping to achieve a cure for chronic hepatitis B infection.

## Introduction

Hepatitis B virus (HBV) infection is estimated to affect approximately 0.8–1.1 % of Canadians [1, 2]. Antiviral treatments approved and available in Canada for chronic hepatitis B (CHB) infection include interferon (standard or pegylated interferon (Peg-IFN)) and nucleos(t)ide analogs (NAs), including an l-nucleoside analog (lamivudine (LAM)), acyclic nucleoside phosphonates (adefovir dipivoxil (ADV) and tenofovir disoproxil fumarate (TDF)), and a d-cyclopentane class nucleoside analog (entecavir (ETV)) [3]. Although most Canadian provinces and territories provide some measure of health plan reimbursement or coverage for these treatments, there may be strict clinical restrictions imposed for reimbursement, especially for more expensive second-generation NA (i.e., TDF and ETV), which limits their use and effectiveness [4–6]. Treatment is normally restricted to CHB carriers in a prolonged immune-active phase of infection or with advanced liver disease. In both HBV e antigen (HBeAg)-positive and HBeAg-negative patients, clinicians assess on-treatment response to NA by monitoring serum HBV DNA (i.e., suppression of viral replication), antibody to HBeAg (anti-HBe) seroconversion (in HBeAg-positive patients), normalization of serum liver transaminases, and improvement in liver stiffness or histology, as determined by transient elastography (FibroScan**®)** or liver biopsy [7•, 8]. Large randomized clinical trials have demonstrated that achievement of these surrogate clinical endpoints lead to reduced risk of liver disease progression, hepatocellular carcinoma (HCC) development, and even fibrosis regression [9, 10]. In contrast, the assessment of response to Peg-IFN therapy is mainly based on off-treatment virological and clinical markers. Thus, a “sustained virologic response” (SVR) to Peg-IFN therapy is defined as HBeAg loss (in HBeAg-positive patients), HBV DNA <2000 IU/mL, as well as persistent normalization of alanine aminotransferase (ALT) at 6 months after the end of treatment. HBsAg loss is rare but more likely to occur with Peg-IFN compared to NA treatment [11]. As discussed below, recent data indicate that quantitative hepatitis B surface antigen (qHBsAg), as well as HBV DNA testing, could predict SVR to Peg-IFN therapy and NA. Table 1 describes the summary of current international guidelines for NA cessation and definitions of treatment response and relapse following withdrawal.

**Table 1:** Summary of current international guidelines for NA treatment cessation

Guideline	HBeAg (+)	HBeAg (−)
CASL/American Association for the Study of Liver Diseases (AASLD)	Non-cirrhotic - Anti-HBe seroconversion - Stop NA after consolidation (Normal ALT and DNA neg × 1–3 years) - Monitor q 3-month × 1 year - OR Rx until HBsAg loss Cirrhotic - Continue Rx - May consider if HBsAg loss - Monitor ALT monthly	Non-Cirrhotic - Continue until HBsAg loss - Monitor ALT q 3 months × 1 year Cirrhotic - Consider if HBsAg loss - Risk flares unknown - Monitor ALT monthly
EASL	- Similar to AASLD - Only if Rx with potent second-generation NA (TDF, ETV) - Durable off-treatment response in 40-80 %	- Similar to AASLD - Continue until HBsAg loss - Indefinitely in cirrhotic
APASL	- Stop 1 year—ideally 3 years after HBeAg seroconversion	- continue until HBV DNA neg × 3, 6 months apart

Definitions: viral relapse: HBV DNA >2000 IU/mL after stopping Rx in patients with virological response. Clinical relapse: viral relapse along with ALT >2 × ULN or baseline ALT. Sustained response: HBV DNA <200 IU/mL and ALT normal after stopping therapy. Complete response: sustained off-treatment virological response, together with loss of HBsAg

This review will provide a comprehensive overview of clinical virological tests used in monitoring response to antiviral therapy for CHB infection, including the current assays for HBV DNA, more recent novel assays such as qHBsAg, and potential monitoring assays (Table 2).

**Table 2:** Summary of prognostic and monitoring laboratory tests for directing management of HBV

Test^a^	Detection target	Interpretation and comments
Hepatitis B e antigen (HBeAg)	Soluble protein produced during acute or chronic infection	Indicates active viral replication and high infectivity. Used to assess likelihood of chronic hepatitis and HBV carriage. HBeAg status indicates HBV infection phase and is used to optimize treatment strategies.
Anti-hepatitis B e antibody (Anti-HBe)	Antibody produced in response to the hepatitis B e antigen	Indicates convalescence or seroconversion/treatment response in previously HBeAg-positive patients
Quantitative hepatitis B surface antigen (qHBsAg)	HBV protein produced during infection; either virion-associated, subviral, or via integrated viral DNA in the host genome.	qHBsAg varies with HBV genotype and infection phase and should be used together with HBV DNA levels for interpretation of results. Absolute qHBsAg values (IU/mL) and rate of decline can be used for response prediction.
Quantitative anti-hepatitis B core antibody (qAnti-HBc)	Antibody produced in response to the hepatitis B core antigen (HBcAg)	Indicator of past, resolved, or persistent HBV infection. Baseline anti-HBc levels may indicate the magnitude of host immune response against HBV, predicting response to therapy. qAnti-HBc testing is not routinely used in management and requires international standardization.
Quantitative hepatitis B corerelated antigen (qHBcrAg)	Core antigen associated with circulating viral particles, denatured HBeAg and the p22 core-related protein	qHBcrAg is correlated with HBV DNA in HBeAg-negative inactive patients and with cccDNA levels. qHBcrAg is negatively predictive for PEG-IFN response. Requires standardization and further study for use as a management tool.
Quantitative hepatitis B viral DNA	Hepatitis B viral genetic material	HBV viral load, ALT, and HBeAg status together with fibrosis state establish treatment initiation and procedures. qHBV DNA is used to monitor and manage therapy and post-therapy and predict cirrhosis and HCC development.
Hepatitis B virus resistance mutations (HBV polymerase gene)	Mutations in the HBV polymerase associated with resistance to: LAM (L80V/I, V173L, L180M, A181T/V, M204V/I); ADV (A181T/V, N236T); ETV^b^ (T184S/A/I/L/G/C/M, S202C/G/I, M250I/V); and TDF^c^ (A181T/V, N236T)	Select/modify appropriate treatment in patients who have been treated previously or in those who are not responding to treatment
Basal core promoter (BCP) and precore (PC) gene region mutations	The most common basal core promoter mutation, A1762T/G1764A, and the precore stop codon mutation, G1896A	These mutations may influence immune response and disease outcome. G1896A and A1762T/G1764A abolish and reduce expression of HBeAg, respectively. Both mutations are associated with different HBV genotypes, and A1762T/G1764A is significantly associated with an increased HCC risk.
HBV genotyping	Complete HBV genome or the HBsAg coding region	Identify HBV genotype (A–H) for epidemiologic and prognostic purposes. Certain HBV genotypes have been shown to respond better to PEG-IFN therapy or to be associated with more severe disease outcomes.

^a^All tests use plasma or serum

^b^Resistance to ETV occurs in the presence of L180 and M204 mutations together with a combination of the listed mutations

^c^Mutations may result in reduced susceptibility. Clinical resistance to TDF has not been reproducibly demonstrated

## HBV DNA monitoring

Monitoring of virological, serological, and clinical markers of infection during treatment and following its withdrawal is essential in evaluating treatment efficacy and patient response. Monitoring of serum HBV DNA has long been considered the best viral marker for management of chronic infection, due to the fundamental role of HBV DNA levels in disease progression and the persistent suppression of HBV DNA as a reliable endpoint goal of antiviral therapy [12•]. Long-term population-based studies have demonstrated the importance of serum HBV DNA levels in predicting the risk for development of severe end-stage liver disease outcomes [13]. Sensitive monitoring of HBV DNA during treatment has also established that stable suppression of HBV DNA to low or undetectable levels improves histologic outcomes and reverses fibrosis or cirrhosis [8, 14]. Treatment management has been facilitated by improvement in HBV DNA quantitative technologies, allowing for increased sensitivity and dynamic range. Most clinical laboratories utilize commercially available real-time polymerase chain reaction (PCR) methods having sensitive limits of detection and quantification for measuring serum HBV DNA; however, other technologies, such as digital PCR, are being investigated as alternative methods, although standardization, ease of use, and optimization will be required to match the convenience and sensitivity of real-time PCR [15, 16].

### HBV DNA LoD and undetectable HBV DNA

Assay sensitivity, which determines the limit of HBV DNA detection and quantification, differs among commercial assays; thus, the definition of “undetectable DNA” in the literature and management guidelines will vary. Prior to the development of an HBV DNA standard IU for quantification, genome equivalents or copies were used, often resulting in significant variability among assays [17, 18]. Due to this variability and use of assays having lower limits of detection, the findings of earlier studies investigating treatment response and cessation may not be comparable to later studies involving sensitive real-time PCR assays [19, 20].

Most management guidelines define virological response as “undetectable HBV DNA as measured by a sensitive PCR assay” [21, 22]. Practically, this translates into a range of values based on the detection limits of quantitative real time HBV PCR assays [23•]. Prior to 2007, most commercially available quantitative HBV DNA assays had a relatively narrow linear range and limit of detection (LoD) of approximately 200 IU/mL. Current real-time PCR assays have an 8 log_10_ linear range with very low LoD: <6 IU/mL (Roche Cobas High Pure/Cobas TaqMan), <10–15 IU/mL (Abbott m2000, dependent on the input volume), and <20 IU/mL (Roche Cobas AmpliPrep/Cobas TaqMan) [24–26] (Table 2). However, we often observe that at the analytical LoD of the Roche Cobas High Pure/Cobas TaqMan assay, single specimen results may fluctuate between “target not detected” (TND) and “<6.0 IU/mL” upon duplicate testing (data not published). The cost of these assays is substantial, and as a result, samples are rarely duplicate tested; therefore, quantification results close to the LoD should be interpreted with caution. Despite an observed high correlation between Roche and Abbott HBV DNA quantification assays, discrepancies have been described in patients undergoing antiviral therapy, such that 25/134 (18.7 %) NA-treated patients had no detectable HBV DNA by the Roche assay but were persistently positive with the Abbott test [27, 28]. These differences may be indirectly associated with the effect of long-term NA therapy and selective pressures upon the viral genome [29]. We have also observed discrepancies in the HBV DNA level, especially in samples having <6.0 IU/mL as detected by the Roche Cobas High Pure/Cobas TaqMan assay, and were able to link these differences directly to nucleotide variation within the region of the reverse primer used in the quantification assay (data not published) [30].

Management guidelines do specify that “assays of lower sensitivity are not recommended” for monitoring during therapy; however, it is unknown whether use of “more” sensitive HBV DNA assays would be clinically relevant. A correlation between the level of HBV DNA at the time of treatment cessation, based on the limit of detection of the assay used in the study, and the risk for relapse has not been fully investigated. However, it is clear that relapse rates remain highly variable after controlling for differing definitions of virological relapse and viral load LoDs defining undetectable DNA [31]. For example, studies by Chi et al. and Seto et al., investigating HBeAg-negative patients discontinuing NA therapy, used <200 and <20 IU/mL, respectively, as the definition of undetectable DNA. Chi et al. observed 54 % of patients to relapse after 1 year, while Seto et al. observed 91.4 % of patients to relapse within 48 weeks [32••, 33]. The variation observed in these and numerous other studies, all of which followed suggested management guideline endpoints, illustrates the lack of association between an undetectable HBV DNA IU/mL value and risk of relapse. However, differences in study design and population, duration of therapy or consolidation, and different endpoint definitions likely contribute to the variability observed [31].

### Role of residual HBV DNA at treatment cessation

Despite this lack of association, the clinical relevance of minimal residual viremia at the time of treatment cessation and during off-treatment follow-up is still a question of interest. Few studies have looked at viral rebound after NA treatment achieved HBV DNA inhibition below the PCR LoD, defined as TND or <10 IU/mL by the most sensitive assays. Undetectable HBV DNA in Chinese NA-treated patients was investigated in a retrospective study, which re-analyzed samples using a highly sensitive PCR assay and reported residual HBV viremia in 41.7 % with a significantly higher detectable rate and mean level of HBV DNA in the relapse group compared to the non-relapse group [34]. Another study involving 120 Chinese patients receiving ETV treatment reported a similar rate of residual HBV viremia—34.2 % [35]. Contrary to these studies, residual HBV viremia was present in 94 % of German patients who were previously presumed to have undetectable HBV DNA (LoD 60–80 IU/mL) after NA treatment. Upon re-testing the samples with a sensitive real-time PCR (LoD <10 IU/mL; Abbott), continuously detectable residual HBV DNA was demonstrated in one third of the patients in all samples throughout the treatment (11–111 months), while the rest had complete suppression of the viral replication in at least one of several consecutive samples [36]. It should be noted that while the majority of the samples were positive with values lower than the LoD of the previous PCR assay, some had values well above that LoD. The median residual viremia levels among HBeAg-positive and HBeAg-negative patients were 16 (1–197) and 9 (1–549) IU/mL, respectively. Although such discrepancies are not unusual considering the expected higher variation with minimal residual viremia, this makes it more difficult to determine which marginal level of virus replication may be associated with viral rebound or sustained response. Considering the lack of association with HBV DNA levels (<200 IU/mL) and relapse as described earlier, further residual DNA studies with larger patient populations will be needed to confirm this finding or determine what role trace HBV DNA has in virological relapse.

### Quantitative HBsAg monitoring of treatment response to Peg-IFN

Standardized qHBsAg assays have only recently become commercially available (Table 2), and they cannot distinguish between the different forms of HBsAg produced either virion associated, subviral forms or produced from integrated sequences [37, 38]. Serum qHBsAg appears to be more strongly correlated with intrahepatic HBV covalently closed circular DNA (cccDNA) levels in HBeAg-positive patients compared to HBeAg-negative patients. This is possibly due to more viral integration events as well as the presence of more virus-associated HBsAg in highly viremic HBeAg-positive patients. Our studies and others have shown that the qHBsAg varies with HBV genotype and with CHB disease phase [39–41]. The levels are highest in the immune tolerance phase, with a decline during the immune clearance phase and a slow progressive decrease after HBeAg seroconversion [42, 43••]. Recent studies suggest that a single qHBsAg measurement (<1000 IU/mL) along with HBV DNA <2000 IU/mL is predictive for maintenance of inactive carrier status and risk of reactivation (96 % positive predictive value), reflecting improved host immune control of HBV infection (Table 3) [44].

**Table 3:** Guidelines for interpretation of quantitative hepatitis B surface antigen testing

Disease phase	Expected baseline range	Annual decline	Comments
	Log_10_IU/mL	Log_10_IU/mL (ethnicity)	
Inactive (HBV DNA is <2000 IU/mL)	1.5–3.0	0.043–0.077 (Asian)	∼3.3 log_10_ (i.e., 1000–<2000 IU/mL inactive, Mediterranean/European) 1.69–2 log_10_ (i.e., <50–100 IU/mL in Asians) predicts HBsAg loss at 6 years
Immune-tolerant	4.5–5.0	0.006 (Asian)	∼5 log_10_ (i.e., >100,000 IU/mL) indicates immune tolerance. Can be used to differentiate immune clearance if HBV DNA is high and ALT is minimally elevated.
HBeAg-negative/reactivation	∼3.0	–	Lower baseline HBsAg predicts decline and loss. Higher baseline level predicts HBeAg-negative hepatitis flare.
NA-treated	<2 log_10_ (100—1000 IU/mL lower risk of relapse after NA stopped (Asians)	Decline >0.5 log_10_ in 2 years after achieve HBV DNA suppression assoc. HBsAg loss (Western European) or 0.166 log_10_ IU/ mL/year (Asian)	Rapid decline >1 log_10_ after 1 year predicts HBsAg loss. Limited effect on qHBsAg
Peg-IFN	HBeAg-positive: - Week 12 HBsAg >20000 IU/mL (∼4.3 log_10_) = non-response (Asians) - Week 12 <300 IU/mL and 24 <1500 IU/mL (3.1 log_10_) predicts SVR - Week 24 non-responder if no HBsAg decline from baseline and <2 log drop in HBV DNA (genotype D) HBeAg-negative: - Week 12 decline >0.5 log_10_—high response - Week 12 decline + 2 log drop HBV DNA ∼40 % develop SVR - <10 IU/mL at week 48 predicted HBsAg loss		HBsAg decline on Rx is genotype-specific A > B, D > C, E.
12 week stopping rules			
			In HBeAg-negative CHB, qHBsAg may be used to help predict response.

qHBsAg may vary in individual patients and require serial monitoring. The qHBsAg should be used together with HBV DNA for interpretation of results. Published data include studies with genotype A (Africans, North America, European, HIV, IDU); genotypes B, C (Asian); genotype D (Mediterranean and Eastern Europe); and genotype E (Africans). The published data on immune-active qHBsAg levels are very variable and are not included

Expert management guidelines for CHB recommend that Peg-IFN be considered a first option for treatment in selected patients due to the advantages of a finite duration of therapy and the greater likelihood of durable HBeAg and HBsAg seroconversions [21, 45]. In HBeAg-positive patients, a standard duration of a 48-week therapy induces approximately ≥30 % HBeAg seroconversion, while the same course of therapy is recommended in HBeAg-negative patients having independent predictors of response, such as younger age and female gender [46, 47]. As Peg-IFN treatment is also associated with adverse effects, other predictors of response, such as HBV genotype and pre-treatment HBV DNA and ALT levels, have been developed to help select those patients best suited for Peg-IFN treatment [48–50]. Based on this data, a useful Peg-IFN HBV treatment index to predict an SVR has been developed (www.liver-GI.nl/peg-IFN) [50].

**Fig. 1:**
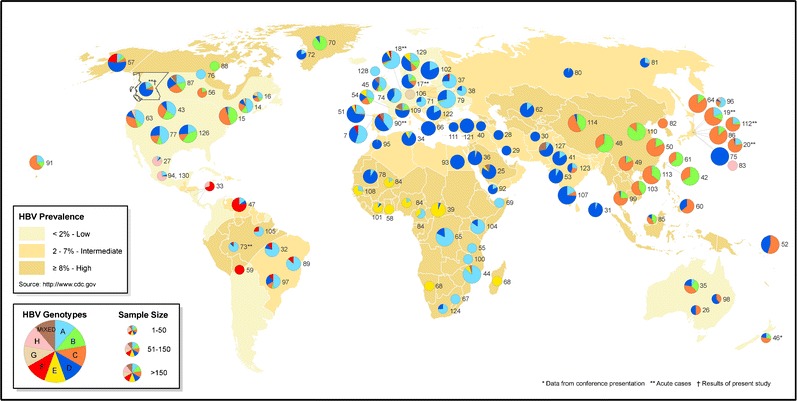
Global geographical distribution of HBV genotypes. The map of genotype distribution and prevalence was determined through literature review and published in a previous study of acute hepatitis B infection in British Columbia, Canada [99]. The figure is reproduced with permission from John Wiley and Sons.

HBV genotype determination is also an important aspect of Peg-IFN treatment response prediction and all management guidelines recommend HBV genotyping among patients being considered for IFN therapy, yet many jurisdictions do not offer this service [7•, 21, 22]. The ten HBV genotypes (A–J) have a distinct geographic distribution and the natural history of each genotype contributes to the major transmission mode and associated endemicity often observed in different regions of the world [51]. A map showing the global geographical distribution of HBV genotypes is shown in Fig. 1. Genotype A was demonstrated to be an independent risk factor for the progression to chronic infection following transmission and acute infection, possibly due to the increased transmission rate and persistent viremia that has been described for genotype A [52–54]. Despite increased chronicity among genotype A-infected individuals, genotype A is associated with an increased likelihood of HBsAg seroclearance compared to other genotypes, particularly in patients treated with Peg-IFN [50]. Similarly, genotype B-infected individuals have demonstrated a higher likelihood of stable HBsAg seroclearance compared to genotype C-infected patients [55].

**Fig. 2:**
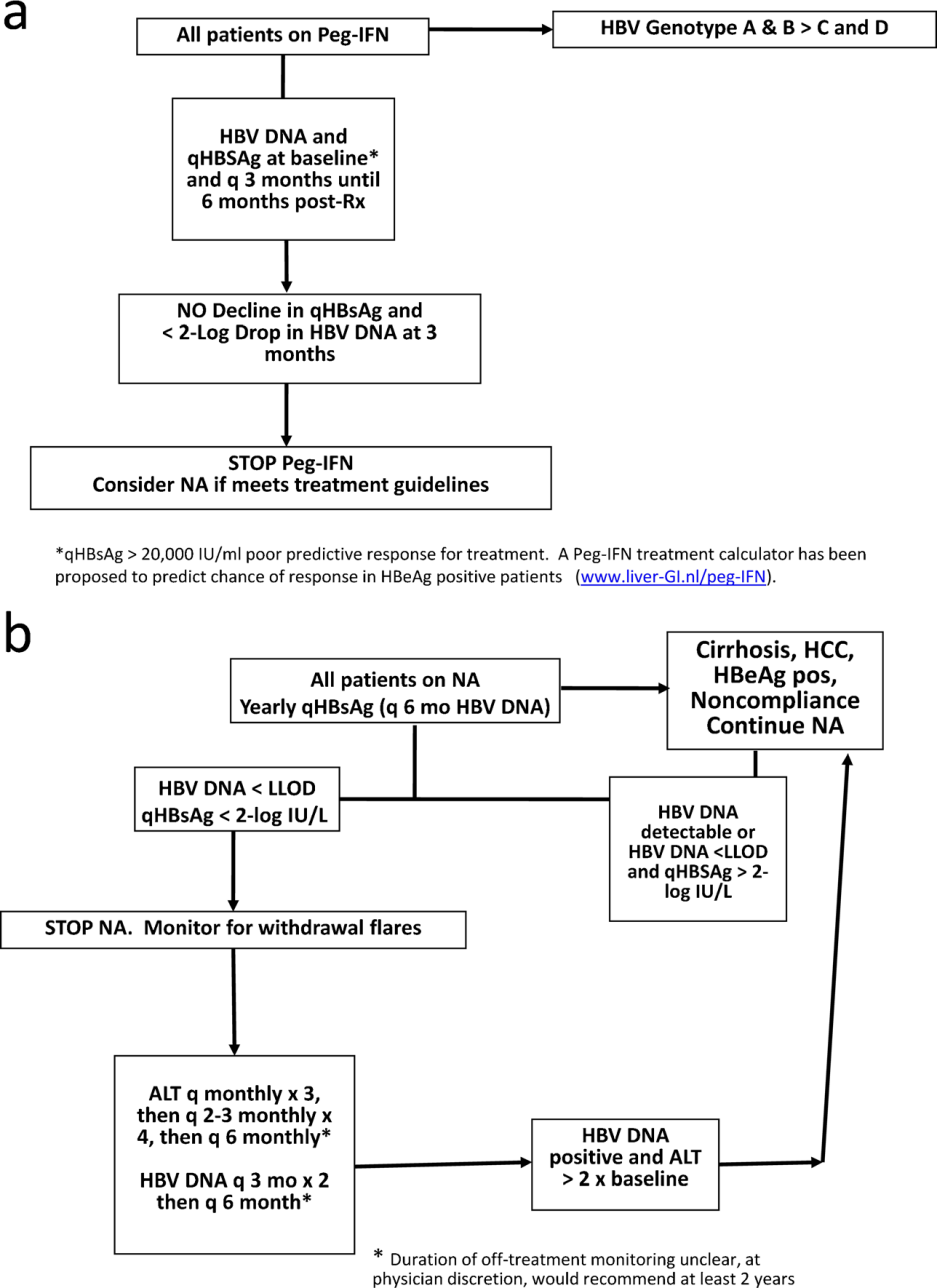
**a** Proposed clinical algorithm for qHBsAg monitoring during Peg-IFN therapy and early stopping rule at 12 weeks for prediction of a sustained virologic response (*q* quantify). **b** Proposed clinical algorithm for cessation of long-term NA therapy based on qHBsAg monitoring and HBV DNA testing according to a sensitive clinical PCR assay (*q* quantify).

Quantitative HBsAg levels have recently been shown to provide an accurate prediction of response to Peg-IFN. Baseline qHBsAg levels <20,000 IU/mL have been correlated with achievement of SVR at 6 months post-treatment [56]. The decline in qHBsAg is particularly useful as an on-treatment biomarker and negative predictive stopping rule [57]. Thus, a lack of qHBsAg decline by week 12 and a <2-log_10_ decline in HBV DNA indicates subsequent non-response (95–100 % negative predictive value) in both HBeAg-positive and HBeAg-negative patients (Table 3) [58–60]. A proposed algorithm for using qHBsAg to guide response to Peg-IFN treatment is provided (Fig. 2a).

### Quantitative HBsAg monitoring of treatment response to NAs

Oral NAs directly inhibit HBV reverse transcriptase activity during infection and so have the advantage of rapidly reducing HBV replication resulting in reduced HBV DNA levels and normalization of serum ALT. Current management guidelines recommend either ETV or TDF as an initial therapy for the treatment of naïve patients due to their potency and high genetic barrier to resistance [21, 22, 61]. NA treatments are not considered to readily “cure” or eradicate HBV infection, due to the persistence of the HBV replication cycle intermediate, cccDNA, which is not targeted by NAs [62]. Long-term or indefinite treatment is recommended in order to reach secondary endpoints, such as HBeAg seroconversion and undetectable HBV DNA, at which time treatment cessation may be considered (Table 1).

A more rapid HBsAg decline in the first year after starting NA or a reduction rate of >0.166 log_10_IU/mL/year may predict subsequent HBsAg loss [63]. In studies involving Asian patients, a qHBsAg level <2 log_10_IU/mL predicted lower risk of increased virological and biochemical responses after stopping NA therapy with 93 % probability of achieving an off-treatment SVR [38, 64–66]. In European patients, with HBV genotype D infection, qHBsAg titres <1000 IU/mL could predict remission and a subsequent HBsAg loss [67]. Although differences in inclusion criteria limited comparison across studies, NA-treated HBeAg-positive patients show greater qHBsAg decline compared to HBeAg-negative patients, especially with higher baseline ALT levels [68]. As noted in our studies and others, there are genotype-specific differences in HBsAg kinetics in response to NA therapy; thus, more studies in regions with a multiethnic genotype distribution, such as Canada, are needed to confirm the results of Asian studies [39, 41, 69]. Further long-term prospective evaluation is also needed regarding the impact of baseline HBsAg levels and the kinetics of qHBsAg decline in identifying patients who will have a durable off-treatment NA response. A proposed algorithm for using qHBsAg to guide response to NAs is provided (Fig. 2b).

### Discontinuation of NA therapy: rules

Understanding the factors necessary for an enduring clinical response to allow the discontinuation of NA treatment is critical, as lifelong therapy has potential drawbacks, such as maintaining compliance and ongoing financial costs [65, 70]. In order for treatment cessation to be considered, defined stopping rules have been suggested based on clinical studies over the past several years. It is agreed that patients having decompensated cirrhosis, regardless of HBeAg status, are excluded from finite treatment options, due to the risk of liver failure if virological rebound occurs [7•].

Studies involving ETV and TDF have contributed to current recommendations guiding NA treatment cessation in HBeAg-positive patients. Treatment discontinuation is suggested for those patients achieving HBeAg seroconversion together with sustained undetectable HBV DNA, based on two consecutive measurements at least 6 months apart, and ALT normalization following at least 1 year but preferably 3 years of consolidation therapy [71, 72•]. Extended consolidation (>3 years) provided a substantially lower relapse rate compared to <1-year consolidation for both HBeAg-positive (25 vs. 54 %; *P* = 0.063) and HBeAg-negative patients (24 vs. 57 %; *P* = 0.036) [32••].

The HBeAg-negative patient population does not have a specific endpoint for treatment cessation; thus, most management guidelines recommend lifelong treatment, until sustained HBsAg loss or seroconversion is achieved [21, 22, 61]. However, the Asian-Pacific Association for the Study of the Liver (APASL) guidelines have suggested that discontinuation of treatment may be considered in HBeAg-negative patients, following at least 2 years treatment and having documented undetectable HBV DNA (<12 IU/mL) on 3 separate occasions at least 6 months apart [72•]. Similarly, other associations acknowledge that undetectable HBV DNA (based on a sensitive PCR assay) and ALT normalization may be considered practical measures of response in HBeAg-negative patients [61]. Indeed, undetectable HBV DNA is achievable in a larger percentage of HBeAg-negative patients compared to HBeAg-positive patients, largely due to their lower baseline DNA levels [73]. However, studies that followed APASL guidelines for HBeAg-negative treatment cessation describe high rates (>80 %) of virologic relapse within 48 weeks post-ETV treatment [33, 74]. In such cases, early virological relapse characterized by low HBV DNA levels (<2000 IU/mL) in the absence of a marked ALT flare may be an acceptable or even desirable scenario, allowing for host immune control to suppress viral replication instead of immediately initiating rescue therapy [75, 76••].

### Novel virological/serological tests for monitoring treatment response

Aside from qHBsAg, there is much interest and urgency in finding more effective tools to predict treatment response prior to and during treatment, particularly for NAs. Other non-invasive serological markers, such as HBV core-related antigen (HBcrAg) and quantitative anti-HBc (qAnti-HBc), have been investigated for their utility in monitoring the natural history of infection and response to treatment (Table 2). Both biomarkers hold promise for predictive therapeutic management.

A chemiluminescent enzyme immunoassay for quantitative measurement of HBcrAg in serum (Lumipulse G HBcrAg, Fujirebio, Gent, Belgium), which detects core antigen associated with circulating viral particles, as well as denatured HBeAg and the p22 core-related protein, following detergent pre-treatment, provides a surrogate measure of the level of transcription and translation of the precore/core coding region within hepatocytes [77, 78]. This is reflected in the positive correlation between HBcrAg and HBV DNA levels in HBeAg-negative inactive patients and with cccDNA levels [79–81]. In this regard, increasing HBcrAg levels over time have been shown to be an independent risk factor for developing HCC [82]. Several studies have demonstrated a risk of relapse following NA treatment withdrawal with HBcrAg levels >3.4–3.7 log_10_U/mL, such that lower HBcrAg levels either before treatment initiation or at the end of treatment correlated with therapeutic response [83–86, 87•]. As absolute HBcrAg levels are reduced in the presence of precore or basal core promoter mutants due to eradication of HBeAg expression, supplementary mutation testing of patients may be required to fully understand individual patient results and ultimately for HBcrAg to be used as a management tool [81, 86]. Although several studies have shown a moderate correlation between HBcrAg and qHBsAg levels, one study has shown a greater negative predictive likelihood with week 12 HBcrAg values (>8.0 log_10_U/mL) as compared to week 12 qHBsAg levels (>4.3 log_10_U/mL) at 94.4 and 80.0 %, respectively, for predicting response to Peg-IFN treatment [79–81]. Thus, monitoring of HBcrAg levels may offer a more robust predictor of virological response during therapy and potential development of HCC.

Quantitative anti-HBc levels can be measured using a commercially available immunoassay (Wantai, Beijing, China) and have been found to be a sensitive and specific indicator of the host immune response. Anti-HBc production is significantly higher during the immune clearance phase and during HBeAg-negative active infection, as compared to the immune tolerant or low replicative phases of infection. Similarly, qAnti-HBc levels are significantly associated with liver enzyme levels during the immune clearance phase [88–90]. Several studies have investigated the utility of baseline qAnti-HBc to predict response to treatment. In studies of patients treated with Peg-IFN, patients who seroconverted from HBeAg positivity to anti-HBe positivity, following end of treatment or follow-up, had significantly higher levels of qAnti-HBc at baseline. Furthermore, qAnti-HBc levels at baseline were considered to be the best independent predictor of HBeAg seroconversion following either Peg-IFN or telbivudine/ADV treatments compared to the baseline predictive value of ALT or HBV DNA levels, with a predictive threshold value of qAnti-HBc at baseline proposed (≥4.4–4.5 log_10_IU/mL) [91, 92]. Using qAnti-HBc as a surrogate marker of the overall immune response may allow for more effective prediction of achieving response to therapy and has been shown to be independent of HBV genotype, unlike qHBsAg, and to have a longer half-life in serum, as compared to ALT [90, 93].

## Conclusions

Over the past decade, clinical studies and research investigations have provided an improved understanding of how best to use current antiviral therapies for HBV. However, until new treatments are developed for novel targets associated with HBV infection, such as cccDNA, host immune effectors, modified direct-acting antivirals, or viral entry, assembly or secretion inhibitors, management of patients on current antiviral therapies will continue to require careful monitoring and laboratory testing [94–98]. The virological monitoring tests, including HBV serological markers, quantitative HBV DNA, and qHBsAg, continue to improve in terms of sensitivity and linear range. Highly sensitive HBV DNA assays should be used to determine the association and level of residual viremia with relapse, and whether a TND quantification result is essential for complete suppression of viral replication and SVR. Increasing data from clinical studies indicate that both HBV genotyping and qHBsAg monitoring may not be available in most clinical diagnostic labs yet may be useful for predicting CHB natural history and monitoring on-treatment response, and hence, should be available to expert providers. Potential serological markers under development offer the prospect of increased predictive value for treatment response and will complement present monitoring methods.
